# Geniposide Protects Against Myocardial Infarction Injury via the Restoration in Gut Microbiota and Gut–Brain Axis

**DOI:** 10.1111/jcmm.70406

**Published:** 2025-02-05

**Authors:** Jie Chen, Tong Zhu, Jinbao Yang, Mengqing Shen, Danmei Wang, Boyuan Gu, Jin Xu, Mingxia Zhang, Xiuli Hao, Zheng Tang, Jie Tong, Yan Du, Bao Zhang, Hongbao Li, MengLu Xu

**Affiliations:** ^1^ The Affiliated Xi'an International Medical Center Hospital Northwest University Xi'an Shaanxi China; ^2^ College of Forensic Medicine Xi'an Jiaotong University Health Science Center Xi'an Shaanxi China; ^3^ Department of Nephrology The First Affiliated Hospital of Xi'an Medical University Xi'an Shaanxi China; ^4^ Department of Physiology and Pathophysiology Xi'an Jiaotong University School of Basic Medical Sciences Xi'an China

**Keywords:** geniposide, gut microbiota, myocardial infarction, paraventricular nucleus, short‐chain fatty acids, sympathetic hyperactivity

## Abstract

Improving gut dysbiosis and impaired gut–brain axis has been a potent therapeutic strategy for treating myocardial infarction (MI). Geniposide (GEN), a traditional Chinese medicine extract, has demonstrated substantial cardioprotective properties post‐MI. Nevertheless, the effect of GEN on gut microbial, gut–brain communication, and its potential mechanism remains unclear. In this study, we initially found that GEN significantly alleviated MI‐induced cardiac dysfunction from echocardiographic data and decreased myocardial fibrosis, inflammation, apoptosis and hypertrophy post‐MI. Additionally, we investigated the effects of GEN on gut pathology, and observed that GEN led to a remarkable change in gut microbiota as evidenced by altering β‐diversity and short‐chain fatty acids (SCFAs) levels, and alleviated intestinal damage indicated by reduced inflammation and barrier permeability post‐MI. Finally, our investigation into brain pathology revealed that GEN induced a remarkable inhibition in PVN inflammation and sympathetic activity following MI. Collectively, these findings imply that the cardioprotective effects of GEN against MI were mediated possibly via an improvement in the impaired gut–brain axis. Mechanically, GEN‐induced increase of microbiota‐derived SCFAs might be the critical factor linking gut microbiota and reduced neuroinflammation with PVN, which leads to the suppression of sympathetic activation, therefore protecting the myocardium against MI‐induced damage.

## 1 Introduction

Myocardial infarction (MI), usually referred to as a heart attack in lay terms, is always caused by a reduction or stoppage of blood flow to the heart, leading to persistent damage to the myocardium. The common symptoms of MI patients include chest pain, dyspnea, excessive sweating, dizziness, muscular weakness and depression, and MI is characterised by high hospital readmission rates and mortality. Although percutaneous coronary intervention (PCI) is widely applied in clinical treatment to re‐establish blood flow, the reperfusion itself might further deteriorate cardiac injury [1]. In addition, several drugs, such as nitrates, antithrombotic agents and angiotensin‐converting enzyme (ACE) inhibitors, have limited effectiveness in treating MI due to their short half‐life, side effects and restricted drug distribution [2]. Thus, it is critical to find new strategies to improve the prognosis of MI patients. Recently, accumulating evidence revealed a close association between gut microbiota and the severity of MI [3]. For example, Dong et al. reported that MI patients exhibited specific changes in gut microbiota composition and serum/urinary/faecal metabolites [4]. Additionally, a report by Tang et al. showed that gut microbiota depletion affects the immune system, worsens heart function and induces high mortality post‐MI [5]. Collectively, modulating gut microbiota composition might be a promising therapeutic strategy to attenuate myocardial injury and exert cardioprotective effects after MI.

Geniposide (GEN), a principal bioactive constituent isolated from a traditional Chinese herb *Gardenia jasminoides* , has been demonstrated to exert extensive pharmacological activities, such as anti‐inflammatory, anti‐apoptosis and anti‐oxidative stress actions [6]. Recently, mounting evidence has supported the cardio‐protective effects of GEN in treating cardiovascular diseases [7]. Zhang et al. reported that GEN could reduce myocardial injury and improve cardiac function by inhibiting autophagy post‐MI [8]. In addition, the therapeutic benefits of GEN against MI have also been verified by Shi et al., and inhibition of NLRP3 inflammasome‐mediated pyroptosis might be the potential pathological mechanism [7]. However, the effects of GEN on gut microbiome post‐MI were not fully elucidated. Interestingly, Hu et al. reported that GEN could induce a remarkable change in gut microbiota composition for treating Non‐alcoholic fatty liver disease (NAFLD), and depletion of gut bacteria could reverse the protective effect of GEN, indicating the critical role of gut microbiota in the therapeutic mechanism of GEN treatment [9]. Thus, these observations raised the possibility that the therapeutic effects of GEN post‐MI were mediated via regulating gut microbiota.

It has been recognised that MI‐induced excessive inflammation within the paraventricular nucleus (PVN) plays a vital role in activating sympathetic hyperactivity [10]. In addition, sympathetic hyperactivity contributes a lot to fatal ventricular arrhythmias (VAs) and sudden cardiac death following MI, and rebalancing the cardiac autonomic system is considered a fundamental therapeutic approach to improve the prognosis of MI patients [11]. Furthermore, the gut–brain axis, a bidirectional communication between the gut and brain, has gained emerging attention among researchers, suggesting that gut microbes play a crucial role in shaping the progression of brain development or dysfunction [12]. More importantly, our previous research has found that altered gut microbiota could affect neuroinflammation in the PVN and sympathetic activity [13]. However, whether the alterations of the gut microbial community induced by GEN could reduce the inflammatory and sympathetic activity in PVN post‐MI remains unclear. Therefore, this research aims to investigate the gut microbiota alterations and their role and mechanisms of rebalancing dysfunctional gut–brain axis under GEN treatment post‐MI.

## 2 Materials and Methods

### 2.1 Animals

All male C57BL/6J mice (8–10 weeks, weighting 25 ± 2 g) were obtained from Xi'an Jiaotong University Animal Laboratory. All mice were randomly housed in a controlled environment (23°C ± 2°C, 55% ± 5% relative humidity) under a 12‐h light and dark cycle. Standard laboratory water and food were available ad libitum. Five mice were housed per cage and acclimatised to the laboratory conditions for a week prior to the experiment. All the animal experiments were approved by Xi'an Jiaotong University Laboratory Animal Administration Committee and were carried out strictly in accordance with Xi'an Jiaotong University Guidelines for Animal Experimentation [14].

### 2.2 MI Model

The MI mice model was created by ligating the left anterior descending (LAD) coronary artery, as previously reported [15]. Under sterile conditions, the mice were anaesthetised and maintained in narcosis by inhaling 2% isoflurane at a flow rate of 2 L/min. They were then positioned on their backs on an operation board. A skin incision was made along the lower edge of the left pectoralis major muscle of the left upper limb. A blunt thoracotomy was performed in the third to fourth intercostal space. Then, the LAD coronary artery was ligated with a 6–0 suture needle at about 2 mm below the pulmonary artery conus, followed by repositioning the heart. After repositioning the heart and compressing the chest to remove air from the cavity, a 3‐0 suture needle was used to suture the thoracic cavity and skin. During the operation, electrocardiogram (ECG) was conducted, and the successful ligation was confirmed by a ST‐segment elevation along with the paleness of anterior wall of the left ventricle. After waking up, the mice were extubated and observed for 15 min. The Sham mice were subjected to the same surgery without ligation.

### 2.3 Experimental Design

The mice were randomly assigned to four groups (*n* = 216): Sham group (*n* = 54), GEN group (*n* = 54), MI group (*n* = 54) and MI + GEN group (*n* = 54). GEN (purity > 98% as determined by HPLC analysis) was obtained from Sigma‐Aldrich (#SML0153) [16]. In the Sham group, mice received oral administration of 0.9% saline (0.2 mL/ day) for 60 days following thoracotomy surgery without ligation. On the final day of the 60‐day period, echocardiography assessments were conducted before the mice were killed. In the GEN group, mice were treated with GEN (50 mg/kg/day) dissolved in 0.9% saline (0.2 mL) via intragastric administration for 60 consecutive days after undergoing thoracotomy surgery without ligation [16]. On the last day of the 60‐day period, echocardiography was performed, and the mice were subsequently killed. In the MI group, mice received daily administration of 0.9% saline (0.2 mL) for 60 days after MI. On the final day of the 60‐day period, echocardiography was conducted, followed by the killing of the mice. In the MI + GEN group, mice were administered GEN (50 mg/kg/day, 0.2 mL) intragastrically for 60 consecutive days post‐MI. On the last day of the 60‐day period, echocardiography assessments were performed before the mice were killed. Regarding the oral gavage technique, a reusable, straight, 20‐gauge stainless steel feeding needle with a 2.25‐mm ball was employed to deliver 0.2 mL sterile 0.9% saline or GEN solution. To ensure that the ball tip of the gavage needle passed the entire length of the oesophagus, an 8.0‐cm‐long gavage needle was utilised. The needle was never forcibly advanced or pushed beyond the premeasured distance from the nose tip to the costal margin (4.5 cm) [17].

### 2.4 Histological Analysis

At the final day of the 60‐day period post‐MI, mice were euthanized by cervical dislocation, and the heart, proximal colon and PVN tissues were quickly removed and cleaned by phosphate‐buffered saline (Solarbio). Then, the tissues were fixed in 4% paraformaldehyde followed by embedded with paraffin. Sections were serially sectioned at 6‐μm thickness. The slices (6 μm) were incubated with Haematoxylin–Eosin (HE) and Masson's trichrome staining as reported previously [18]. The stained tissue slices were imaged under a light microscope (Nikon, Japan) to detect the histopathological alterations and cardiac fibrosis. For WGA staining, heart tissues were rapidly frozen in OCT at −80°C and then sliced into 10 μm thickness. Then, slices were incubated with WGA‐FITC (sigma # L4895) according to the manufacturers' instruction as previously described. The WGA‐stained slices were observed under a confocal laser microscope (Nikon, Tokyo, Japan) [15].

For heart tissues: Masson's trichrome staining was conducted to evaluate the cardiac fibrosis [15]. HE staining was performed to observe the morphological characteristics of cardiac tissue [15]. Masson staining was used to detect the infarct scar size [19]. WGA staining was used to measure the cross‐sectional area of cardiomyocytes [15]. Staining of TNF‐α and IL‐1β was used to measure the inflammation response in the cardiac tissues [20]. Co‐staining of CD68 and α‐actinin was detect the macrophages infiltration in the cardiomyocytes [21]. Co‐staining of tunel and α‐actinin was to detect the apoptotic cardiomyocytes in the cardiac tissues. Co‐staining of tunel and vimentin was to detect the apoptotic fibroblasts in the cardiac tissues. Co‐staining of Tunel and CD31 was to detect the apoptotic endothelial cells in the cardiac tissues [22]. CD31 staining was used to measure the capillary density, and α‐SMA staining was used to measure the arteriolar density in the cardiac tissues [23].

For proximal colon tissues: HE staining was utilised to assess the crypt length, the number of crypts and the thickness of the muscle layer. Masson's trichrome staining was conducted to analyse the fibrotic area. ZO‐1, Occludin, and Cingulin staining were performed to evaluate intestinal barrier function. TNF‐α and IL‐1β staining was used to assess the inflammatory responses [24].

For PVN tissues: Iba‐1 staining was utilised to determine the proportion of activated microglia. TNF‐α and IL‐1β staining assessed the pro‐inflammatory response, while IL‐10 staining detected the anti‐inflammatory response. Tyrosine hydroxylase (TH) staining was employed to measure the extent of sympathetic activity [13].

For hippocampus tissues: To measure the volume of the dentate gyrus (DG), sections were stained with DAPI. The hippocampal areas of interest were outlined and measured using ImageJ software. The DG volume for each mouse was determined by summing all traced areas and multiplying by the section thickness and a spacing factor of 6. To quantify the GFAP expression level, GFAP staining was conducted. Images were first converted to 8‐bit format, and three regions of interest (ROIs) were selected per section: DG, CA1 or CA2/3. The mean grey value of each ROI was calculated and normalised using the corresponding value obtained from the same ROI in a control image taken from the thalamus of the same section. The normalised values of the three ROIs for each mouse were then averaged to determine the mean GFAP expression value [25].

### 2.5 Heart Weight/Body Weight

At the end of the experiment, the mice were anaesthetised with isoflurane. Then, the heart was quickly excised, washed with phosphate‐buffered saline (Solarbio), and heart weight was measured. The ratio of heart weight/body weight (HW/BW) was calculated to represent the level of cardiac hypertrophy [26].

### 2.6 Stool Collection, DNA Extraction, Library Preparation and Sequencing

Faecal samples from six mice per group were collected freshly before the mice were executed. These samples were squeezed into an empty sterile tube and immediately stored at −80°C for subsequent sequencing analysis as previously described. The total genomic DNA from faecal samples was extracted using Zymoresearch Faecal/Soil DNA isolation kit (Zymoresearch, Irvine, CA). After detecting the DNA concentration with NanoDrop One (Thermo Fischer, USA), an ABI GeneAmp 9700 PCR thermocycler was used to amplify the hypervariable V3‐V4 region of the bacterial 16S rRNA gene. The PCR reactions were conducted in triplicate. All PCR products were extracted from 2% agarose gel, and subjected to gel purification (Axygen Biosciences, USA) prior to quantification by using Quantus Fluorometer (Promega, #E615), according to the manufacturer's instructions. Each amplicon was pooled equally. Pooled library was checked for quality using a 2100 Bioanalyzer (Agilent, Santa Clara, CA) before sequencing. Finally, the purified amplicons were sequenced on an Illumina Miseq system (Illumina, USA). The Quantitative Insights in Microbial Ecology (QIIME II) software package (version 2021.11) was applied to process the sequencing results. The amplicon sequence variants (ASVs) were subsequently picked using QIIME II, and taxonomy assignment was performed using Silva (version 138.1) as the reference database. Bacterial alpha diversity indices Chao1 (an indicator of species richness) and Shannon diversity were calculated for each sample using QIIME alpha rarefaction command. The alpha diversity inter‐group differences were analysed by Wilcoxon test with FDR adjusted *p* value. The alpha diversity was calculated for each sample using the R package Vegan and beta diversity is presented as 3D ordination plots based on unweighted UniFrac principal coordinate analysis (PCoA) [27]. Permutational MANOVA (PERMANOVA) test was used to determine significant differences between two groups of samples.

### 2.7 Echocardiography

Echocardiographic analysis was performed according to a previously described protocol [28]. After being anaesthetised with isoflurane, mice were put on a heating plate and then subjected to detection of cardiac function using Echocardiography equipment system (Visual Sonic Vevo 3100, Toronto, ON, Canada). The heart rate (HR) and ECG signals were recorded. Two‐dimensional M‐mode images were obtained to detect the left ventricular systolic functional parameters such as left ventricular internal diameter diastolic (LVIDd), left ventricular internal diameter systolic (LVIDs), left ventricular end‐diastolic volume (LVEDV) and left ventricular end‐systolic volume (LVESV). Left ventricular fractional shortening (LVFS) and left ventricular ejection fraction (LVEF) were measured using the Vevo 3100 software. To assess the regional systolic wall thickening, the LV end‐diastolic thickness and LV end‐systolic thickness in both border and remote zone were measured using the Vevo 3100 software, and the systolic wall thickening (%) was calculated as 100 × (LV end‐systolic thickness−LV end‐diastolic thickness)/LV end‐diastolic thickness [29]. Left ventricular diastolic functions were assessed using apical 4‐chamber views. Mitral flow velocities, including peak early (E) and atrial (A) velocities, were obtained from Doppler‐mode echocardiograms. Additionally, early diastolic (E') and atrial (A') velocities of the mitral annulus were measured using Tissue Doppler‐mode echocardiograms. The ratios of E/A, E/A', and E/E' were calculated using the Vevo 3100 software. All these parameters were measured at least three times.

### 2.8 Western Blotting

The mouse heart tissues were isolated and homogenised in ice‐cold Radio Immunoprecipitation Assay (RIPA) lysis buffer (Beyotime, Jiangsu, China) containing the protease inhibitors Phenylmethanesulfonyl fluoride (PMSF, Beyotime). The protein concentration was measured by using NanoDrop 2000C (Thermo Scientific, USA). After equal quantities, total proteins were separated on 8% or 10% SDS‐PAGE by electrophoresis, followed by electroblotting onto polyvinylidene fluoride (PVDF) membranes (Millipore). The membranes were blocked in 5% bovine albumin (BSA) in TBST for 2 h at room temperature. Subsequently, the PVFDs were incubated with the following primary antibodies overnight at 4°C: anti‐Bax (1:1000, 50,599‐2‐Ig, Proteintech), anti‐Bcl 2 (1:1000, 68,103‐1‐Ig, Proteintech), anti‐caspase 3 (1:1000, sc‐56,036, Santa Cruz), anti‐TNF‐α (1:1000, 60,291‐1‐Ig, Proteintech), anti‐IL‐1β (1:1000, sc‐52,012, Santa Cruz), anti‐BNDF (1:1000, GB11559‐100, Servicebio), anti‐Trkb (1:1000, 13,129‐1‐AP, Proteintech) and anti‐β‐actin (1:1000, 20,536‐1‐AP, Proteintech). Then, membranes were incubated with the anti‐mouse secondary antibody (1:1000, A0216, Beyotime) or anti‐rabbit secondary antibody (1:1000, A0208, Beyotime) at room temperature for 1 h. The bands were detected using the ECL chemiluminescence system (Clinx Science Instruments, China). Densitometric analysis was performed using NIH Image J software (Bethesda, MD, USA), and data were normalised to β‐actin [30].

### 2.9 Immunofluorescence Staining

The heart, brain and proximal colon slices were washed in phosphate‐buffered saline (PBS) for 5 min, permeabilized in 0.3% TritonX‐100 for 10 min, and blocked with 5% bovine serum albumin (BSA) (Sigma) for 1 h. Then, the slices were incubated with specific antibodies overnight at 4°C. For the heart tissues: anti‐TNF‐α (1:200, 60,291‐1‐Ig, Proteintech), anti‐IL‐1β (1:200, sc‐52,012, Santa Cruz), anti‐CD68 (1:200, GB113109‐100, Servicebio), anti‐α‐actinin (1:200, GB111556‐100, Servicebio), vimentin (1:200, GB111308‐100, Servicebio), anti‐CD31 (1:200, GB120005‐100, Servicebio), and anti‐α‐SMA (1:200, GB111364‐100, Servicebio), and Tunel (1:5:50, G1504, Servicebio). The colon slices were treated with anti‐TNF‐α (1:200, 60,291‐1‐Ig, Proteintech), anti‐IL‐1β (1:200, sc‐52,012, Santa Cruz), anti‐ZO‐1 (1:200, 21,773‐1‐AP, Proteintech), anti‐Occludin (1:200, 27,260‐1‐AP, Proteintech), and anti‐Cingulin (1:200, 21,369‐1‐AP, Proteintech). Brain slices were exposed to anti‐Iba‐1 (1:200, GB11105‐100, Servicebio), anti‐GFAP (1:200, GB12100‐100, Servicebio), anti‐IL‐10 (1:200, GB11534‐100, Servicebio), and anti‐TH (1:200, AB152, Millipore Sigma). Subsequently, the slices were then incubated in 594‐ Goat Anti‐Rabbit (1:100, GB28301, Servicebio) or 594‐Goat Anti‐ Mouse (1:100, GB25303, Servicebio) for 1 h, and washed with PBS three times. Then, the slices were stained with DAPI (1:1000, Beyotime) for 5 min. Tissue slices images were captured with a fluorescent microscope (Nikon, DS‐Ri2) and quantified using NIH Image J software. A confocal laser microscope (Nikon, Tokyo, Japan) was used for imaging [31].

### 2.10 Enzyme‐Linked Immunosorbent Assay (ELISA)

At the final day of the 60‐day period post‐MI, the blood samples were harvested from the abdominal aorta. Prior to blood collection, the mice were anaesthetised using 5% isoflurane to ensure they were in deep anaesthesia and experienced no distress. Once adequately anaesthetised, the mice were positioned in a supine position, and a midline incision was made to access the abdominal cavity. The abdominal aorta, located ventrally in the abdominal cavity, was identified. Sterile forceps were used to gently lift the aorta. A syringe equipped with a sterile needle was then inserted into the aorta to collect blood (0.5–1 mL). For plasma preparation, blood samples were collected in sterile tubes containing an anticoagulant and then centrifuged at 4000 g for 15 min at 4°C. For serum preparation, blood samples were collected in sterile tubes without anticoagulants and subsequently centrifuged at 4000 g for 15 min at 4°C. Norepinephrine (NE) in plasma were determined using a NE ELISA kit (Abnova, Taiwan), according to the manufacturer's guidelines [32]. Serum troponin I (cTnI) levels were determined using a cTnI ELISA kit (Elabscience, E‐EL‐M1203c), while serum creatine kinase‐MB (CK‐MB) was assessed with a CK‐MB Assay Kit (Nanjing Jiancheng, China) [33]. In addition, heart tissues were collected, homogenised in PBS, and then centrifuged at 12,000 g for 20 min. The supernatant was collected to assess malondialdehyde (MDA), superoxide dismutase (SOD), and catalase (CAT) activity according to the instructions provided by the kits (NanJing JianCheng Bioengineering Institute) [34].

### 2.11 RNA Isolation and Relative Quantitative RT‐PCR

Total RNA was isolated from myocardium using Trizol reagent (Invitrogen). cDNA was transcribed using a cDNA Archive Kit (Applied Biosystems). Quantitative real‐time PCR (qRT‐PCR) was performed using an ABI Prism 7900 Sequence Detection System (Applied Biosystems). The primers for ANP were 5′‐AGCCGTTCGAGAACTTGTCTT (forward) and 5′‐CAGGTTATTGCCACTTAGGTTCA (reverse). For BNP, the primers used were 5′‐GAGTCCTTCGGTCTCAAG GC (forward) and 5′‐TACAGCCCAAACGACTGACG (reverse). GAPDH served as an internal control for normalising the expression of RMST and other genes. Gene expression levels were calculated using the 2^−ΔΔCt^ method [15].

### 2.12 Statistical Analysis

All data are presented as the mean ± SD, and *p* < 0.05 was statistically significant. The difference among groups were assessed using either one way ANOVA followed by Tukey's multiple comparisons *post hoc tests*. The survival rate of mice was analysed by Kaplan–Meier methods and compared by log‐rank test. All statistical analyses were conducted using GraphPad Prism 9 software [24].

## 3 Results

### 3.1 GEN Treatment Improved Cardiac Function in MI Mice

**FIGURE 1:**
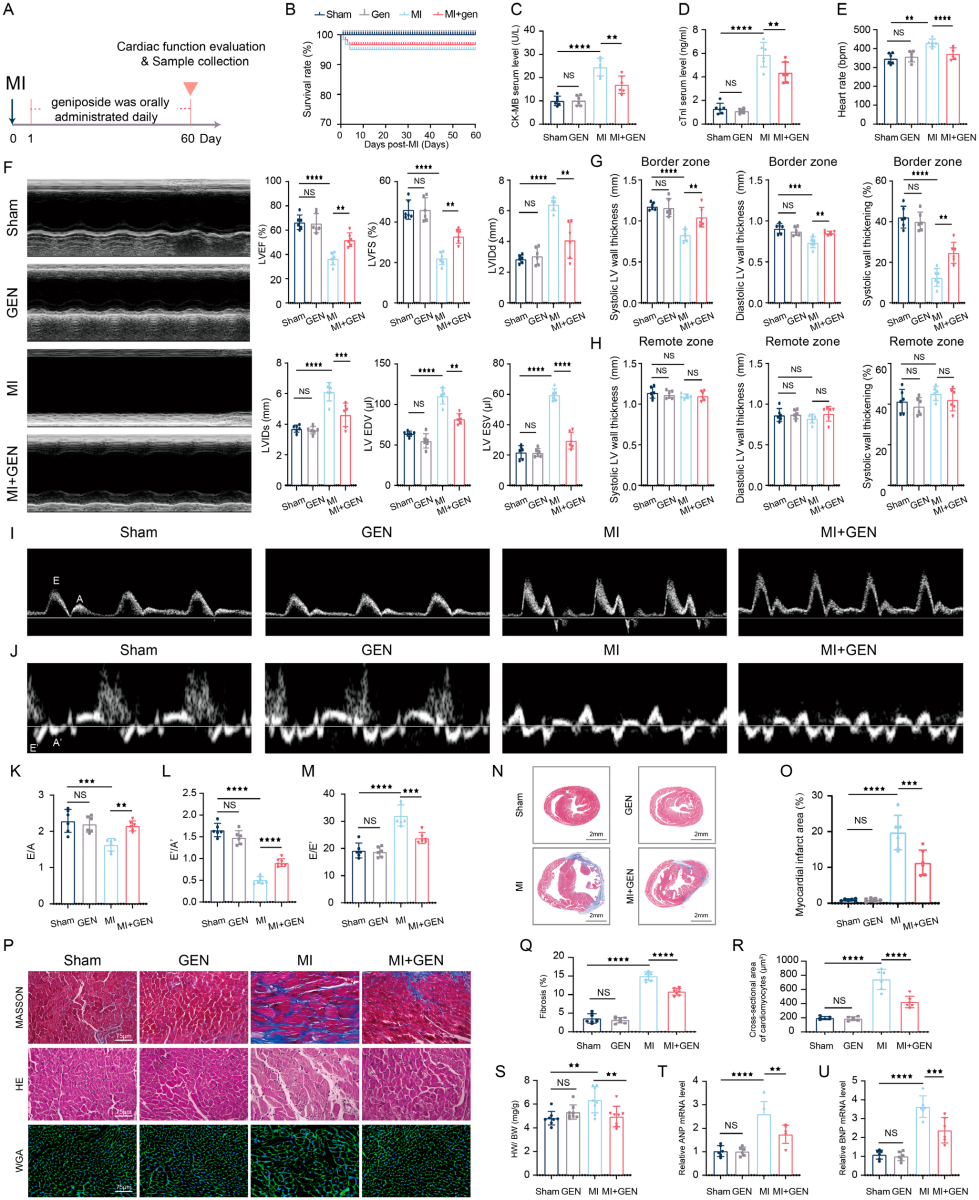
GEN treatment protects against cardiac dysfunction post MI. (A) Schematic illustration of experimental design. (B) Kaplan–Meier curve showing the survival rate among all groups. (C) The CK‐MB levels in serum. (D) The cTnI levels in serum. (E) Quantification of heart rate. (F) Representative M‐mode echocardiography of mice from different groups. Quantification of LVEF, LVFS, LVIDd, LVIDs, LVEDV, and LVESV. (G) Quantification of systolic LV wall thickness, diastolic LV wall thickness and systolic wall thickening in the border region. (H) Quantification of systolic LV wall thickness, diastolic LV wall thickness and systolic wall thickening in the remote region. (I) Representative images of pulsed‐wave Doppler. (J) Representative images of tissue Doppler. (K) Quantification of E/A ratio. (L) Quantification of E'/A' ratio. (M) Quantification of E'/E' ratio. (N) Representative images of Masson staining. (O) Quantification of infarct size. (P) Representative images of left ventricular tissue sections stained with Masson, H&E and WGA. Scale bar = 75 μm. (Q) Quantitative analysis of heart fibrotic area. (R) Quantitative analysis of the cross‐sectional area of cardiomyocytes. (S) Quantitative analysis of heart/body weight. (T) Cardiac ANP mRNA level. (U) Cardiac BNP mRNA level. ANP, atrial natriuretic peptide; BNP, brain natriuretic peptide E/A, ratio of E velocity to A velocity; E'/A', ratio of E' velocity to A' velocity; E/E', ratio of E velocity to E' velocity; HE, haematoxylin–eosin staining; HR, heart rate; HW/BW, heart weight /body weight; LVEDV, left ventricular end‐diastolic volume; LVEF, left ventricular ejection fraction; LVESV, left ventricular end‐systolic volume; LVFS, left ventricular fractional shortening; LVIDd, left ventricular internal diameter diastolic; LVIDs, left ventricular internal diameter systolic; WGA, Wheat germ agglutinin. Data are presented as mean ± SD (*n* = 6) and assessed using one‐way ANOVA followed by Tukey's post hoc test. For all panels: ^★★^ *p* < 0.01, ^★★★^ *p* < 0.001, ^★★★★^ *p* < 0.0001 and ns means not statistically significant.

The ECG results revealed the significant ST‐segment elevation in the MI mice compared to the Sham group, confirming the successful establishment of the MI model (Figure S1). To investigate the effects of GEN on MI‐induced cardiac dysfunction, the mice were orally administrated with GEN daily from 1 to 60 days post‐MI, and then subjected to a series of functional experiments (Figure 1A). All Sham and GEN mice survived until the end of the experimental period. In contrast, MI + GEN group has a higher survival rate compared with MI group (Figure 1B). The testing for MI markers (cTnI and CK‐MB) in serum was carried out, revealing that GEN could suppress the MI‐caused elevation of cTnI and CK‐MB (Figure 1C,D). The HR of mice in each group was measured and it is found that a remarkable rise in HR induced by MI was relieved by GEN treatment (Figure 1E). The echocardiography was performed and showed that MI led to a significant decrease in LVEF and LVFS and a remarkable increase in LVIDd, LVIDs, LVEDV and LVESV, compared with Sham group (Figure 1F). However, GEN treatment could reverse the MI‐induced above changes (Figure 1F). To detect the regional wall function in the left ventricular, the systolic LV wall thickness, the diastolic LV wall thickness and the percent of systolic wall thickening in both border and remote zone were measured.

And, we found that MI induced a significant decrease in the systolic LV wall thickness, the diastolic LV wall thickness and the percent of systolic wall thickening in the border zone, which were partly restored by GEN treatment (Figure 1G). However, no changes in above indexes were observed in the remote region among all groups (Figure 1H). Thus, the above data highlighted the profound effects of GEN on the restoration of cardiac systolic function following MI (Figure 1G,H). Peak early (E) and late (A) blood flow velocities through the mitral valve were quantified using conventional pulsed‐wave Doppler echocardiography (Figure 1I). From the tissue Doppler imaging taken at the septal edge of the mitral annulus, peak early (E') and late (A') diastolic velocities were determined (Figure 1J). MI + GEN mice showed higher levels of E/A and E'/A' and lower levels of E/E', compared to MI group (Figure 1K–M), suggesting that GEN could exert remarkable improvements in the MI‐induced diastolic dysfunction. The Masson staining results revealed that GEN treatment significantly reduced the infarction area, compared with MI group (Figure 1N,O). In addition, Masson staining results also showed that there was a significant decrease in the percentage of fibrotic area in the MI + GEN group, compared with MI group (Figure 1P,Q). And, we found that GEN remarkably reduced the MI‐induced increase of cardiomyocyte cross‐sectional area, as evidenced by the results of HE and WGA staining (Figure 1P,R). Furthermore, we also found GEN alleviated cardiac hypertrophy post‐MI, as evidenced by that heart weight /body weight (HW/BW) and mRNA levels of atrial natriuretic peptide (ANP) and brain natriuretic peptide (BNP) in the MI + GEN group was lower than that in MI group (Figure 1S–U). To conclude, these data consistently suggested that GEN could mitigate cardiac dysfunction after MI.

### 3.2 GEN Mitigated Myocardial Damage by Reducing Inflammation, Apoptosis and Oxidative Stress, While Enhancing Angiogenesis Post‐MI

**FIGURE 2:**
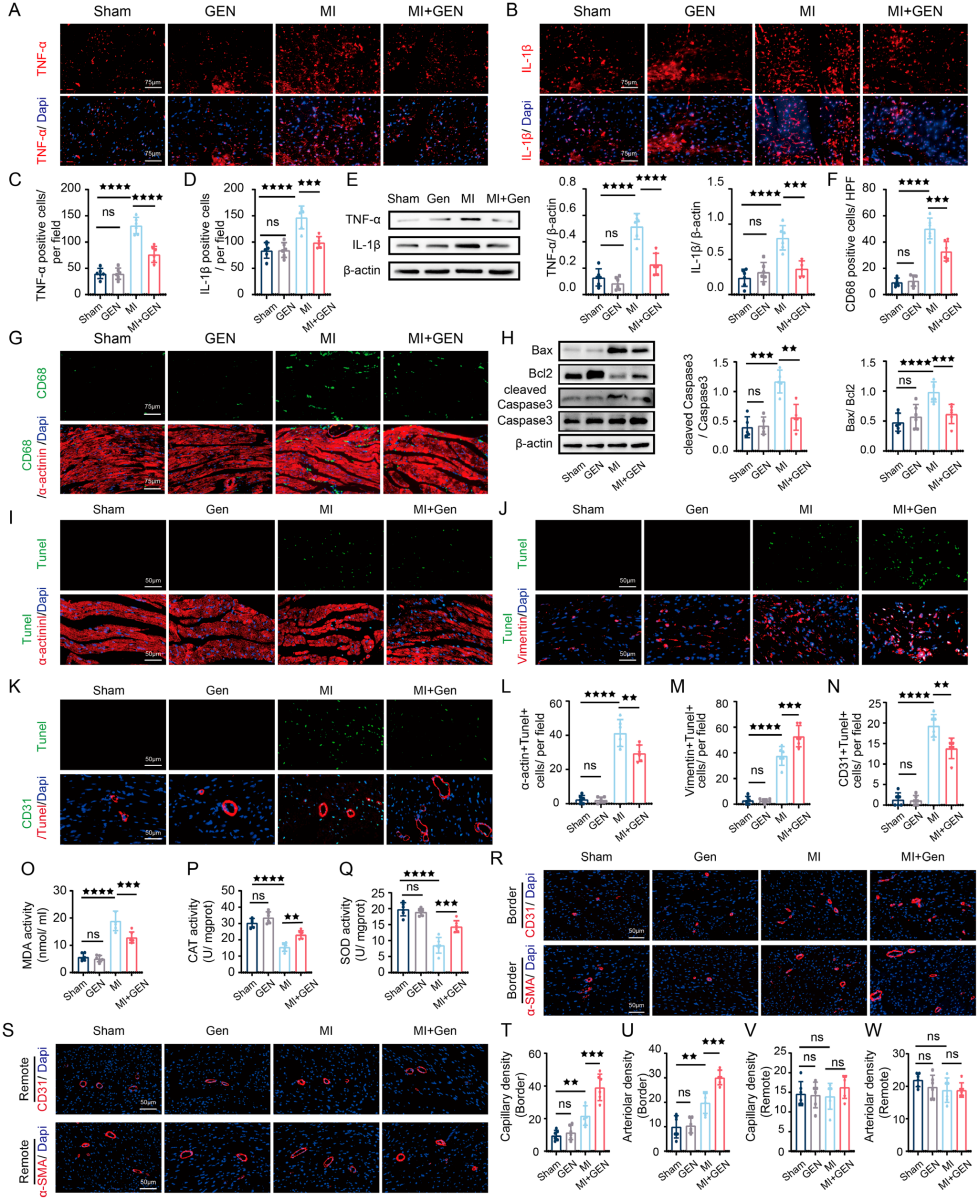
Gen inhibited inflammation, apoptosis and oxidative stress, and enhanced angiogenesis in the cardiac tissues following MI. (A) Immunofluorescence staining of TNF‐α cells. The scale bar is 75 μm. (B) Immunofluorescence staining of IL‐1β cells. The scale bar is 75 μm. (C) Quantitative analysis of TNF‐α positive cells. (D) Quantitative analysis of IL‐1β positive cells. (E) Representative immunoblot and quantitative analysis of TNF‐α and IL‐1β. β‐Actin served as a loading control. (F) Quantitative analysis of CD68 ^+^ α‐actinin ^+^ positive cells. (G) Representative images of double immunofluorescent staining for CD68 and α‐actinin. The scale bar is 75 μm. (H) Representative immunoblot of Bax, Bcl2, cleaved Caspase‐3, Caspase‐3 and β‐Actin. Quantification of Bax/Bcl2 and cleaved Caspase‐3/Caspase‐3. (I) Representative images of double immunofluorescent staining for Tunel with α‐actinin. The scale bar is 50 μm. (J) Representative images of double immunofluorescent staining for Tunel with Vimentin. The scale bar is 50 μm. (K) Representative images of double immunofluorescent staining for Tunel with CD31. The scale bar is 50 μm. (L) Quantitative analysis of α‐actinin ^+^ Tunel ^+^ positive cells. (M) Quantitative analysis of Vimentin ^+^ Tunel ^+^ positive cells. (N) Quantitative analysis of CD31 ^+^ Tunel ^+^ positive cells. (O) The MDA activity. (P) The CAT activity. (Q) The SOD activity. (R) Immunofluorescence staining of CD31 and SMA positive cells in the border zone. The scale bar is 50 μm. (S) Immunofluorescence staining of CD31 and SMA positive cells in the remote zone. The scale bar is 50 μm. (T) Quantitative analysis of capillary density (CD31 positive cells) in the border zone. (U) Quantitative analysis of arteriole density (α‐SMA positive cells) in the border zone. (V) Quantitative analysis of capillary density in the remote zone. (W) Quantitative analysis of arteriole density in the remote zone. Data are presented as mean ± SD (*n* = 6) and assessed using one‐way ANOVA followed by Tukey's post hoc test. For all panels: ^★★^ *p* < 0.01, ^★★★^ *p* < 0.001, ^★★★★^ *p* < 0.0001 and ns means not statistically significant.

To explore the effects of GEN on MI‐induced inflammation, immunofluorescence staining and western blotting for inflammation‐related proteins were performed, and the results revealed that GEN markedly attenuated MI‐caused increase of TNF‐α and IL‐1β in the cardiac tissues, compared to MI group (Figure 2A–E). In addition, double immunofluorescent staining results for CD68 (macrophage marker) of and α‐actinin (cardiomyocyte marker) also demonstrated that macrophages were abundantly infiltrated into myocardium post‐MI, and GEN could induce a notable decrease in the degree of macrophages infiltration in the cardiomyocytes (Figure 2F,G). The expression of apoptosis‐associated proteins was also detected by Western blotting. As shown in Figure 2H, the ratio of Bax/Bcl2 and cleaved Caspase 3/Caspase 3 was significantly reduced by GEN treatment post‐MI. To investigate the specific types of cardiac cells that undergo apoptosis after MI or GEN treatment, we performed double immunofluorescence staining for Tunel with α‐actinin (cardiomyocyte marker), Vimentin (fibroblast marker), and CD31 (endothelial cell marker). The immunofluorescence staining results revealed that a significant increase in the number of α‐actinin ^+^ Tunel ^+^, Vimentin ^+^ Tunel ^+^, and CD31 ^+^ Tunel ^+^ positive cells following MI, indicating the increase of apoptosis among cardiomyocytes, fibroblasts and endothelial cells post‐MI. Importantly, we also found that GEN reduced the number of α‐actinin ^+^ Tunel ^+^ and CD31 ^+^ Tunel ^+^ positive cells, while it increased the number of Vimentin ^+^ Tunel ^+^ positive cells compared to the MI group (Figure 2I–N). These findings suggested that GEN could alleviate apoptosis in cardiomyocytes and endothelial cells, while facilitating apoptosis in fibroblasts following MI (Figure 2I–N). The effects of GEN on oxidative stress of myocardial tissue were also measured, and the results demonstrated that MDA activity of MI + GEN mice was significantly lower than that of MI group, while CAT and SOD activities of MI + GEN group were higher than that of MI group (Figure 2O–Q), indicating the remarkable antioxidant capacity of GEN following MI. Furthermore, CD31 staining was performed to detect the capillary density and α‐SMA was conducted to detect the arteriole capillary. The results of CD31 and α‐SMA staining in the border zone revealed that the CD31 and α‐SMA level were found to be elevated, indicating that the border zone is experiencing the significant vascular remodelling following MI. Then, we also found that GEN increased the number of CD31‐positive and α‐SMA‐positive cells in this region, suggesting that GEN might play a critical role in promoting angiogenesis in the border zone following MI (Figure 2R,T,U). Conversely, in the remote zone, the expression of CD31 and α‐SMA remained unchanged after MI or GEN treatment, suggesting that vascular structure and function in the remote area is relatively unaffected by MI or GEN treatment (Figure 2S,V,W). Collectively, these results demonstrated that GEN might exert cardioprotective effects by inhibiting inflammation apoptosis and oxidative stress, while facilitating angiogenesis post‐MI.

### 3.3 GEN Ameliorated the Pathological Progression of the Intestine Post‐MI

**FIGURE 3:**
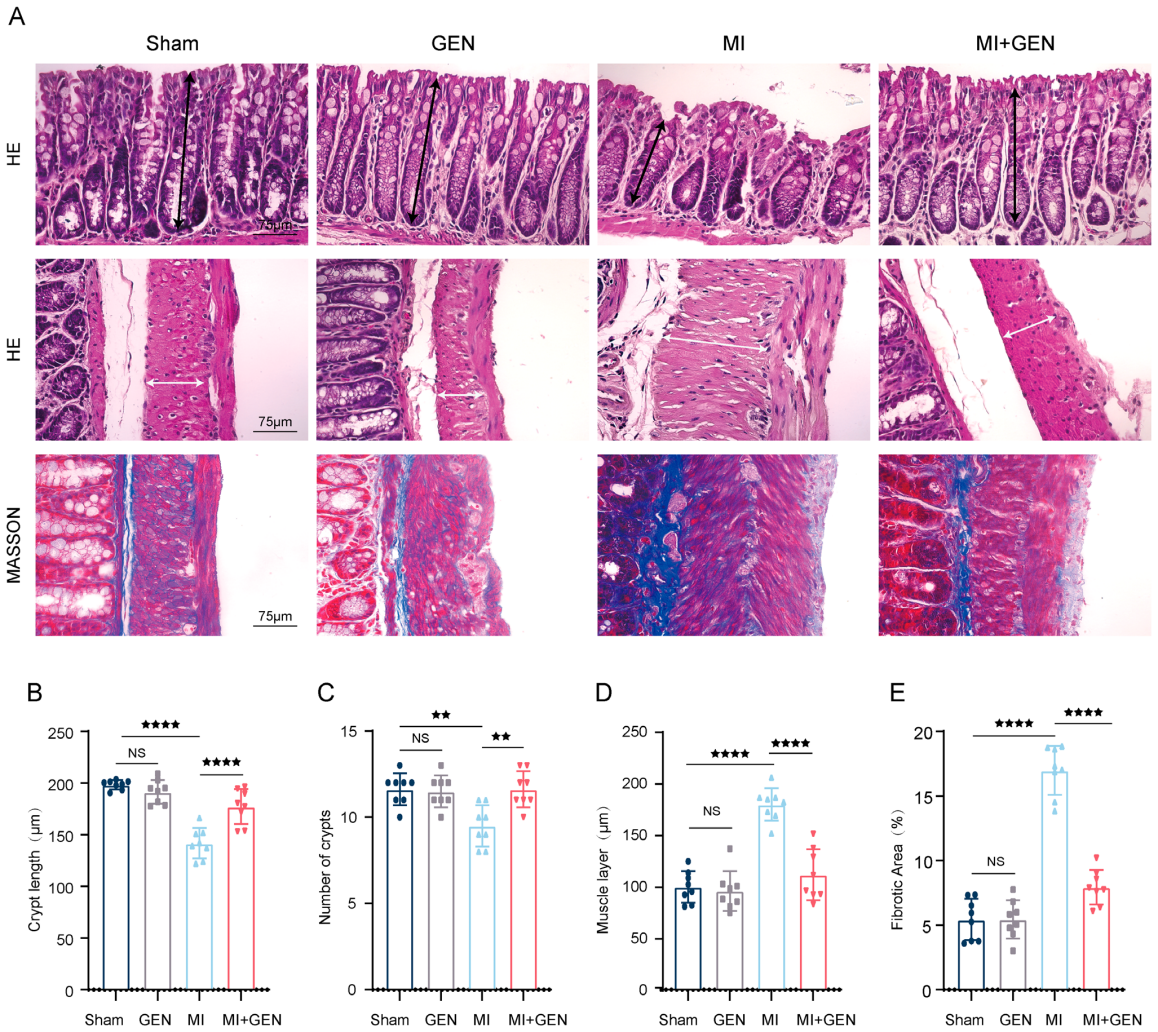
GEN reduced the pathological changes of the gut after MI. (A) Representative pictures of proximal colon sections stained with H&E and Masson from all groups. The scale bar is 75 μm. The black line represents the length of crypt. The white line presents the thickness of muscle layer. (B) Quantitative analysis of crypt length from each group. (C) Quantitative analysis of the number of crypts from each group. (D) Quantitative analysis of the thickness of muscle layer from each group. (E) Quantification of fibrotic area in each group. Data are presented as mean ± SD (*n* = 8) and assessed using one‐way ANOVA followed by Tukey's post hoc test. For all panels: ^★★^ *p* < 0.01, ^★★★★^ *p* < 0.0001 and ns means not statistically significant.

Given the critical role of intestinal pathology post‐MI, we investigated the changes in pathological features of the gut post‐GEN treatment. HE results demonstrated that MI + GEN mice exhibited reduced crypt length and the number of crypts, along with an increased muscle layer thickness, compared to MI mice (Figure 3A–D). However, GEN could effectively reverse the above changes in the gut (Figure 3A–D). In addition, Masson staining was also conducted and revealed that decreased fibrotic area was observed in the MI+ GEN group, compared to that in MI group (Figure 3A,E). Taken together, these data indicated that GEN could remarkably ameliorate gut pathology post‐MI.

### 3.4 GEN Decreased Gut Permeability and Inflammation Post‐MI

**FIGURE 4:**
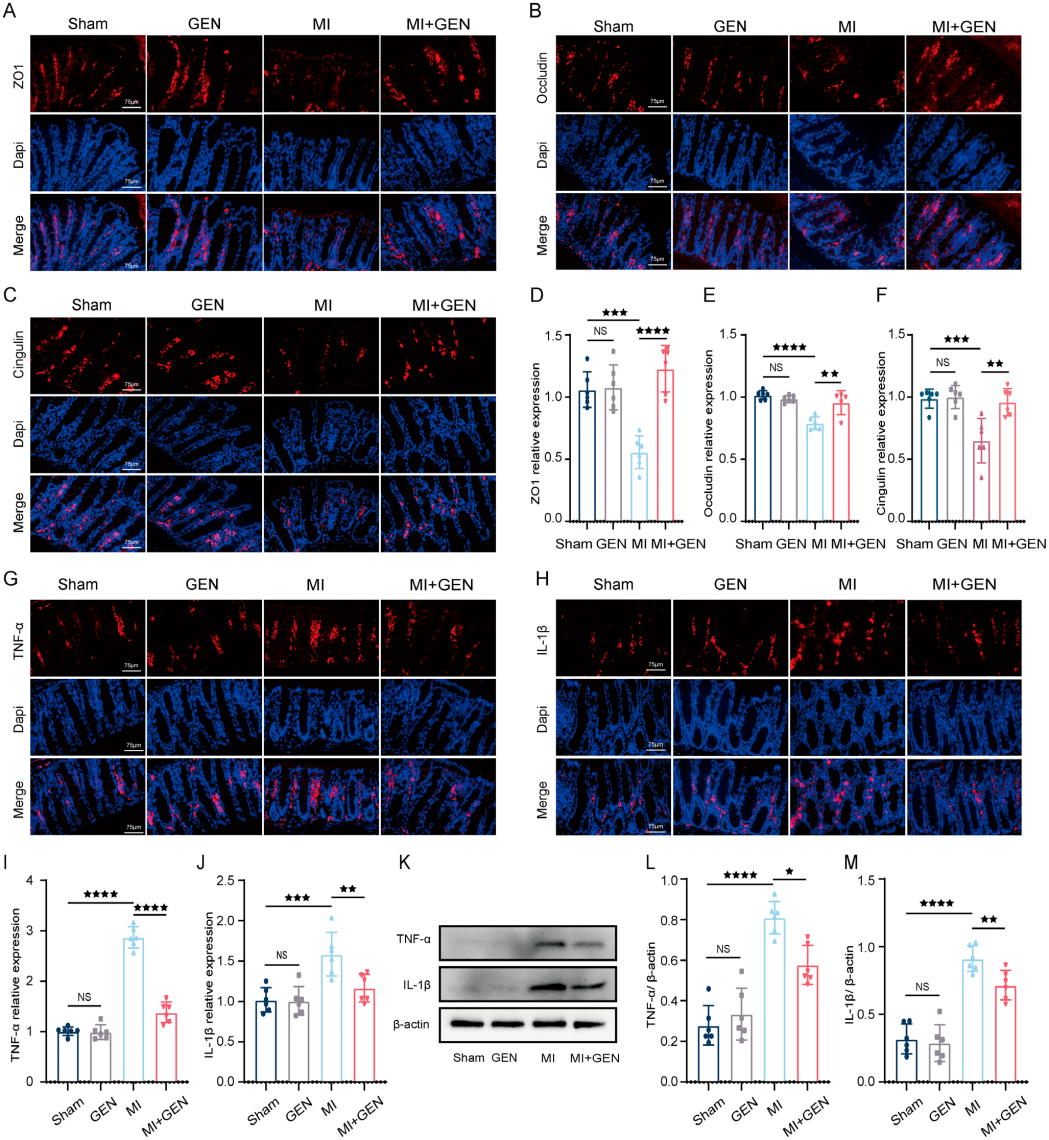
Gen significantly ameliorated gut permeability and inflammation in MI mice. (A‐C) Representative pictures of immunofluorescent staining for ZO1 (A), Occludin (B) and Cingulin (C) in the proximal colon from all groups. Scale bar = 75 μm. (D‐F) Quantitative analysis of ZO1(D), Occludin (E) and Cingulin (F) expression. (G, H) Representative photomicrographs of immunofluorescence staining for TNF‐α (G) and IL‐1β (H) in the proximal colon of each group. Scale bar = 75 μm. (I) The number of TNF‐α positive cells. (J) The number of IL‐1β positive cells. (K) Representative gel bands of TNF‐α, IL‐1β and β‐Actin from each group. β‐Actin was used as a loading control. (L) Quantification of TNF‐α expression from each group. (M) Quantification of IL‐1β expression from each group. Data are presented as mean ± SD (*n* = 6) and assessed using one‐way ANOVA followed by Tukey's post hoc test. For all panels: ^★^ *p* < 0.05, ^★★^ *p* < 0.01, ^★★★^ *p* < 0.001, ^★★★★^ *p* < 0.0001 and ns means not statistically significant.

To determine the effects of GEN on intestinal barrier function after MI, the measurement on levels of intestinal barrier‐related proteins (ZO‐1, Occludin and Cingulin) in the proximal colon was conducted by immunofluorescence staining. The results showed that MI led to a remarkable decrease of ZO‐1 (Figure 4A,D), Occludin (Figure 4B,E) and Cingulin (Figure 4C,F) in the intestinal tissues compared with Sham group (Figure 4A–F), suggesting the increased gut permeability post‐MI. However, the above MI‐induced changes were partly restored by GEN treatment (Figure 4A–F). To investigate the effects of GEN on gut inflammation post‐MI, immunofluorescence staining for several inflammatory cytokines (TNF‐α, IL‐1β) in the proximal colon was performed. The results revealed that MI group exhibited increased expression of TNF‐α and IL‐1β in the gut, which were reversed by GEN treatment (Figure 4G–J). In agreement with immunofluorescence results, western blotting results also showed that GEN significantly reduced MI‐induced increased levels of TNF‐α and IL‐1β in the intestinal tissues (Figure 4K–M). To conclude, these data demonstrated that GEN intervention could significantly protect against intestinal permeability and exhibited an anti‐inflammation capacity in the gut post‐MI.

### 3.5 GEN Altered Gut Microbial Composition in the MI Mice

**FIGURE 5:**
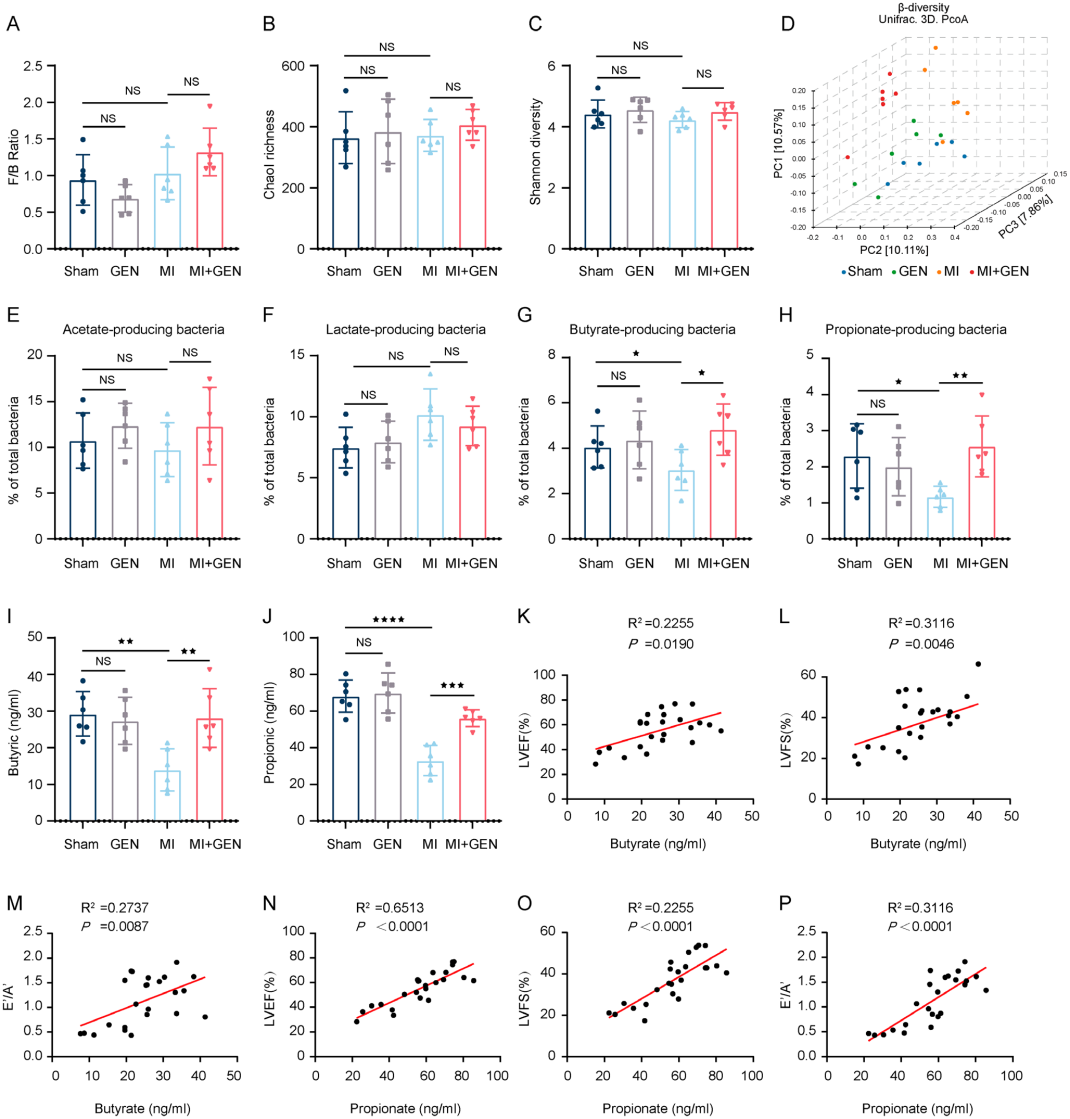
Effects of GEN on the remodelling of gut microbiota post‐MI. (A) The ratio of phyla Firmicutes to Bacteroidetes (F/B). (B) The ratio of Chao1 richness. (C) The ratio of Shannon diversity. α‐diversity indices (Chao1 richness and Shannon diversity) of the gut microbiota were analysed by Wilcoxon test with FDR adjusted *P*‐value. Statistical analysis of Wilcoxon is listed in Tables 1 and 2. (D) The three‐dimensional principal coordinate plot (PCoA) for β‐diversity shows the clustering of gut microbial communities. (E) The level of Acetate‐producing bacteria. (F) The level of Lactate‐producing bacteria. (G) The level of Butyrate‐producing bacteria. (H) The level of Propionate‐producing bacteria. (I) The level of serum Butyric in each group. (J) The level of serum Propionic in each group. (K) Correlation analyses between serum Butyric and LVEF. (L) Correlation analyses between serum Butyric and LVFS. (M) Correlation analyses between serum Butyric and E'/A'. (N) Correlation analyses between serum Propionic and LVEF. (O) Correlation analyses between serum Propionic and LVFS. (P) Correlation analyses between serum Propionic and E'/A'. Data are presented as mean ± SD (*n* = 6) and assessed using one‐way ANOVA followed by Tukey's post hoc test. For all panels: ^★^ *p* < 0.05, ^★★^ *p* < 0.01, ^★★★^ *p* < 0.001, ^★★★★^ *p* < 0.0001 and ns means not statistically significant.

At the end of the experiment, faecal samples were collected freshly before the mice were executed. These samples were squeezed into an empty sterile tube and immediately stored at −80°C for subsequent sequencing analysis. Due to the critical role of gut microbiota in the progression of MI, 16S rDNA sequencing were performed to investigate the composition of the gut microflora in the intestine of the MI mice with or without GEN treatment. There was an increase trend in Firmicutes/Bacteroidetes ratio (F/B), an essential marker of gut microbiota dysbiosis, in the MI + GEN group compared to MI group, though it was not significant (Figure 5A). In addition, we analysed α‐diversity indices (Chao1 richness and Shannon diversity) of the gut microbiota in all experimental groups by Wilcoxon test with FDR adjusted *p*‐value. As shown in Tables 1 and 2, there was no significant difference in Chao1 richness and Shannon diversity among all groups (Figure 5B,C). For β‐diversity, a principal coordinate analysis (PCoA) plot using the UniFrac distance metric (Figure 5D) showed that the plots of the MI group increased the distance from the Sham group (PERMANOVA, *R* ^2^ = 0.1205, *p* = 0.0217, Table 3). Moreover, the gut microbiota from the MI groups were clearly separated from the MI + GEN group (PERMANOVA, *R* ^2^ = 0.1433, *p* = 0.0026, Table 3). No differences in acetate‐producing and Lactate‐producing bacterial communities were observed among all groups (Figure 5E,F). Though, GEN led to an increase in butyrate‐producing and propionate‐producing bacterial communities, compared with MI group (Figure 5G,H). And, we also found that MI group showed higher levels of plasma butyrate and propionate than Sham group, which was significantly reduced by GEN treatment (Figure 5I,J). Notably, butyrate and propionate are both classified as producers of each type of short‐chain fatty acids (SCFA). Then, we performed correlation analysis between plasma microbiota metabolites (butyric and propionic) and indices of cardiac function (LVEF, LVFS, E'/A') respectively. As shown in Figure 5K–P, there was a positive correlation between butyric with LVEF (*R* ^2^ = 0.2255, *p* = 0.0190), LVFS (*R* ^2^ = 0.3116, *p* = 0.0046) as well as E'/A' (*R* ^2^ = 0.2737, *p* = 0.0087). Moreover, propionic was also positively correlated with LVEF (*R* ^2^ = 0.6513, *p* < 0.0001), LVFS (*R* ^2^ = 0.2255, *p* < 0.0001) and E'/A' (*R* ^2^ = 0.3116, *p* < 0.0001), indicating that the regulation of gut microbiota composition might be involved in the cardioprotective effects of GEN. Collectively, these results suggested that GEN altered the gut microbiota composition post‐MI, as evidenced by the remarkable change in β‐diversity and SCFA level.

**TABLE 1:** The Chao1 richness was analysed by Wilcoxon test with FDR adjusted *p* value.

Group 1	Group 2	*p*	FDR
Sham	GEN	0.6991	0.8389
Sham	MI	0.9372	1
Sham	MI + GEN	0.3095	0.6190
GEN	MI	1	1
GEN	MI + GEN	0.3095	0.3714
MI	MI + GEN	0.9372	0.9372

**TABLE 2:** The Shannon diversity was analysed by Wilcoxon test with FDR adjusted *p* value.

Group 1	Group 2	*p*	FDR
Sham	GEN	0.5887	0.8389
Sham	MI	0.4848	1
Sham	MI + GEN	0.4848	0.6991
GEN	MI	0.1796	0.5389
GEN	MI + GEN	0.0931	0.3714
MI	MI + GEN	0.5887	0.9372

**TABLE 3:** Permutational MANOVA (PERMANOVA) was used to determine significant differences between two groups of samples.

Group 1	Group 2	*p*	*R* ^2^‐value
Sham	GEN	0.0024	0.1464
Sham	MI	0.0217	0.1205
Sham	MI + GEN	0.0018	0.1737
GEN	MI	0.0016	0.1584
GEN	MI + GEN	0.0121	0.1355
MI	MI + GEN	0.0026	0.1433

*Note:* Similarity of each of the pairing groups is shown with *R* ^2^ and *p* values. The bigger *R* ^2^ indicated that there was dissimilarity between groups. *p* < 0.05 represent significance.

### 3.6 GEN Alleviated MI‐Induced Increase of Sympathetic Activity via Inhibiting Neuroinflammation Within PVN and Reducing Astrogliosis in the Hippocampus Post‐MI

**FIGURE 6:**
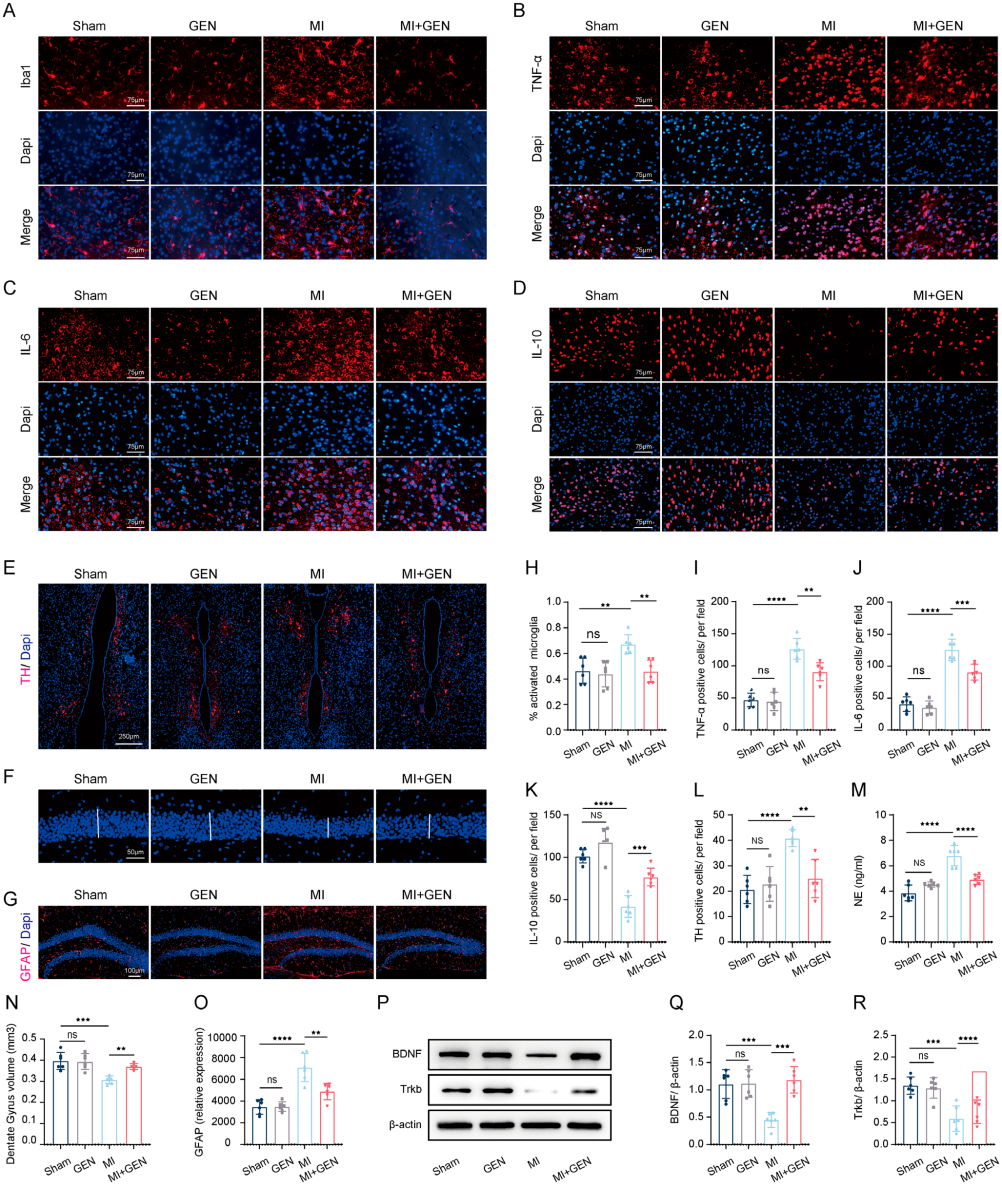
Gen alleviated brain dysfunction by attenuating neuroinflammation in the PVN, reducing astrogliosis in the hippocampus, and inhibiting the sympathetic nerve activity after MI. (A‐D) Immunofluorescence staining for Iba1 (A), TNF‐α (B), IL‐6 (C) and IL‐10 (D) in the PVN from each group. Scale bar = 75 μm. (E) Representative images of immunofluorescent staining for TH in the PVN. Scale bar = 250 μm. (F) Representative images of measurement of dentate gyrus (DG) volume in each group. The white bar denotes the thickness of the DG. Scale bar = 50 μm. (G) Representative images of immunofluorescent staining for GFAP in the DG. Scale bar = 100 μm. (H) The percentage of activated microglia in each group. (I–L) Quantification of TNF‐α (I), IL‐6 (J), IL‐10 (K) and TH (L) positive cells per vision field. (M) The protein level of NE in the serum from each group. (N) Average measure of DG volume. (O) Relative expression of GFAP. (P) Representative gel bands of BDNF, Trkb and β‐Actin from each group. β‐Actin was used as a loading control. (Q) Quantification of BDNF expression. (R) Quantification of Trkb expression. Data are presented as mean ± SD (*n* = 6) and assessed using one‐way ANOVA followed by Tukey's post hoc test. For all panels: ^★★^ *p* < 0.01, ^★★★^ *p* < 0.001, ^★★★★^ *p* < 0.0001 and ns means not statistically significant.

Since the critical role of activated microglia and neuroinflammation within PVN in the pathology progression post‐MI, we detected the effects of GEN on the percentage of activated microglia and expression level of inflammatory factors in the PVN. Specifically, activated microglia exhibit an “ameboid” shape, with larger cell bodies and thickened, shortened processes, whereas non‐activated microglia display a ramified morphology with small cell bodies and long, thin processes. The results of immunofluorescence staining for Iba1 showed that the percentage of activated microglia in the PVN was observed in the MI group compared with that in Sham group, which could be effectively decreased by GEN treatment (Figure 6A,H). Given that activated microglia plays a critical role in the inflammatory progression, we then detected the changes in proinflammatory cytokines (IL‐6 and TNF‐α) and anti‐inflammatory cytokines (IL‐10) using immunofluorescence staining. Compared with MI mice, MI+ GEN mice exhibited a remarkable decrease in the level of IL‐6 and TNF‐α, and a significant increase in the level of IL‐10 (Figure 6B–D,I,J,K). The above results indicated that GEN could significantly reduce microglial activation and neuroinflammation in the PVN post‐MI. Previous studies have established a close association between activated sympathetic activity and increased inflammation within PVN post‐MI. Thus, we tested the expression of several indexes of sympathetic activity (norepinephrine (NE) and TH) from all groups. The results showed that the TH fluorescence intensity in the PVN was remarkably increased in MI group, which was inhibited by GEN treatment (Figure 6E,L). In addition, we also found that GEN led to a significant decrease in the serum NE level, compared with MI group (Figure 6M). Collectively, these data demonstrated that GEN alleviated MI‐induced increased sympathetic activity.

Importantly, previous studies have revealed that the hippocampus serves as a crucial hub for the brain‐heart axis under MI conditions, exerting significant regulatory effects on sympathetic nerve activity [25]. Therefore, we investigated the effects of GEN on hippocampal alterations. First, we observed a reduction in the volume of the dentate gyrus (DG), the main gateway of the hippocampal formation, in the MI group compared to Sham group. The MI‐induced decrease in DG volume was reversed by GEN treatment post‐MI (Figure 6F,N). Then, GFAP‐staining results demonstrated that indicated that GEN significantly reduced astrogliosis in the DG compared to the MI group (Figure 6G,O). Furthermore, western blotting results revealed that GEN significantly increased the levels of BDNF and TrkB in the hippocampus following MI (Figure 6P–R), highlighting GEN's positive regulatory effects on neurogenesis in the hippocampus. In summary, these findings indicated that GEN exerted pronounced neuroprotective effects by reducing neuroinflammation in the PVN, decreasing astrogliosis in the hippocampus, and mitigating sympathetic nerve activity after MI.

## 4 Discussion

**FIGURE 7:**
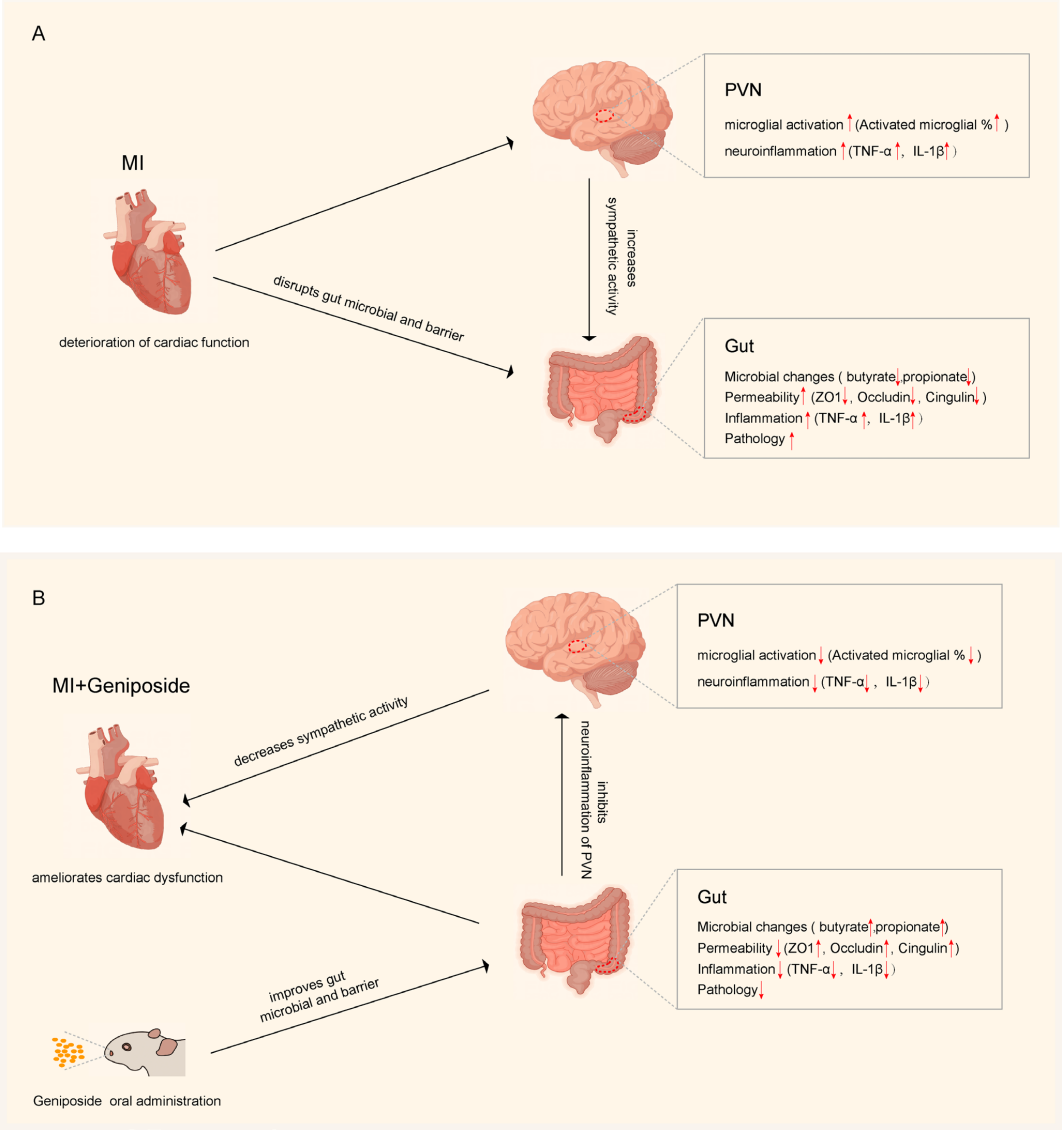
A schematic depicting the possible mechanism of GEN on impaired gut–brain axis post‐TBI. (A) MI induces cardiac dysfunction, increases inflammation with PVN, enhances sympathetic activity, and disrupts the gut microbiota. (B) Oral GEN administration increases the microbiota‐derived SCFAs, and SCFAs might be the factor linking gut microbiota and reduced neuroinflammation with PVN, which leads to the inhibition of sympathetic hyperactivity, therefore exerting cardioprotective effects against MI.

In this study, we established a mice MI model to investigate the effects of GEN treatment on cardiac dysfunction, gut microbiome, neuroinflammation within the PVN, and its potential mechanisms. The significant findings of this study were listed as follows: (1) GEN significantly reduced myocardial fibrosis, inflammation, and apoptosis while enhancing angiogenesis, resulting in a substantial improvement in cardiac function following MI. (2) GEN altered the gut microbiota composition and its corresponding bacterial metabolites and protected against inflammation and permeability in the intestine following MI. (3) Increased neuroinflammation in the PVN, astrogliosis in the hippocampus and hyperactivity of the sympathetic nervous system induced by MI were notably inhibited post‐GEN treatment. Overall, the beneficial effects of GEN could partly be attributed to rebalancing the impaired gut‐brain‐heart axis after MI (Figure 7).

MI, a lethal event of cardiovascular diseases, is often caused by an imbalance in coronary artery blood supply and demand, resulting in myocardial apoptosis, inflammation, and fibrosis, all of which contributes to the cardiac functional and morphological disorders. Recently, more and more attention has focused on the therapy of traditional Chinese medicine for its multitarget characteristics and minor side effects in the treatment of MI disease [35]. GEN, an iridoid extracted from gardenia fruit, has been proven to exert cardioprotective effects via a number of pharmacological properties such as anti‐inflammation, anti‐oxidative stress, anti‐autophagy, and anti‐apoptosis actions [36]. Consistent with previous studies [37], this research demonstrated that GEN significantly ameliorated cardiac hypertrophy, reduced myocardial infarct size and improved cardiac function post‐MI. And, the above cardioprotective effects of GEN against MI were possibly achieved by diminishing myocardial apoptosis, inflammation, and fibrosis, while promoting angiogenesis (Figures 1 and 2). These findings provide robust support for GEN treatment in the future clinical application.

As for the change in inflammatory response, we demonstrated an increase in TNF‐α and IL‐1β levels following MI through western blotting and immunofluorescence assays, and confirmed the infiltration of macrophages in the cardiomyocytes via co‐staining of CD68 and α‐actinin, which were consistent with previous studies [38]. Furthermore, we observed that treatment with GEN effectively inhibited these changes, suggesting a potential therapeutic role in modulating the inflammatory response following MI. Despite these significant findings, we did not specifically determine which cardiac cell types are involved in the inflammatory response and are responsible for the release of these inflammatory cytokines. This gap in understanding is critical, as identifying the specific cell types involved could lead to targeted therapeutic strategies [39, 40]. To address this limitation in future research, several approaches could be considered. First, we intend to employ the single‐cell RNA sequencing to analyse the gene expression profiles of individual cardiac cells, thus providing insights into which cell types are actively participating in the inflammatory response. Second, more specific cell isolation and culture methods could be developed to study the functions of different cardiac cell types separately post‐MI. At the same time, we aim to construct a cell co‐culture model to simulate the in vivo environment of MI, aiming to observe the interaction processes between different cardiac cells such as cardiomyocytes, fibroblasts, and inflammatory cells [41]. By doing so, we can further explore the mechanisms underlying the inflammatory response following MI and potentially identify more targeted therapeutic strategies.

Regarding the alteration in apoptosis level, we found that GEN resulted in the inhibition of apoptosis in cardiomyocytes and endothelial cells, while simultaneously promoting the apoptosis of fibroblasts. This finding suggests that GEN has a selective protective effect on the most critical cell types involved in cardiac function and repair—the cardiomyocytes and endothelial cells—thereby preserving heart tissue integrity and promoting vascular health post‐MI. The inhibition of apoptosis in these cell types is significant, as it may help maintain the structural and functional capacity of the myocardium post‐MI [40, 42]. Conversely, the promotion of fibroblast apoptosis indicates a potential therapeutic strategy for addressing scar formation that can negatively impact cardiac function after MI [43]. By selectively inducing fibroblast apoptosis, GEN may facilitate a more favourable healing response, potentially preventing excessive scarring and promoting a more organised repair process. Thus, these findings highlight the dual role of GEN as a protective agent for vital cardiac cells while simultaneously regulating the survival of fibroblasts;

With respect to the change in angiogenesis level, we observed that vascular regeneration was increased post‐MI and even more pronounced after GEN treatment. The increase in vascular regeneration after MI indicates the heart's natural attempt to restore blood supply to the damaged area. It is a crucial part of the repair process as new blood vessels can bring oxygen and nutrients to the infarcted myocardium, which may help limit the extent of tissue damage and promote the survival of cardiomyocytes [23, 44]. And, the enhanced vascular regeneration after GEN treatment indicates the potential therapeutic effect of GEN in improving the angiogenic process. Significantly, the changes in vascular regeneration occurred in the border zone rather than the remote zone post‐MI. The border zone is a critical area where the infarcted and non‐infarcted tissues meet. The fact that angiogenesis occurs predominantly in this zone reflects the importance of this region in the repair process. The lack of changes in the remote zone suggests that the angiogenic response is localised to the area adjacent to the infarcted region, where the need for increased blood supply is more immediate [45, 46]. This finding also implies that the body's response to MI is targeted and specific, with the focus on the areas that are most affected and in need of repair.

There is an astounding number and diversity of microorganisms in the human gut, referred to as the microbiota. In addition, the vast number of microbes endow the gut microbiota with massive potential for the production of numerous functionally active metabolites, and gut microbiota and host have developed a mutualistic relationship through coevolution, in which their biological interaction plays a critical role in the maintenance of health and disease susceptibility [47]. Notably, an imbalance of gut microbiome richness and biodiversity has been implicated not only in intestinal pathological damage but also in multiple extra‐intestinal diseases, including MI [48]. Recent studies have established a solid association between the gut microbiota and the severity of MI, and the effects of the microbiota and its metabolites on atherosclerosis development and immune modulation might be the possible mechanism [49]. Therefore, gut microbiota has emerged as a potential target for preventing and treating MI. In addition, intestinal pathological conditions can also be considered a critical factor in influencing the gut microbiota. Specifically, MI‐reduced cardiac output leads to increased ischemia, inflammation, and oedema in the intestinal wall, which further compromises the integrity of the intestinal epithelial barrier. And, this cascade of events ultimately contributes to the gut dysbiosis [50]. However, the effects of GEN on gut pathological changes and gut microbiota post‐MI remains largely unclear. In this study, we found that GEN could ameliorate the pathological progression of the intestine following MI. This was evidenced by a reduction in fibrotic area, diminished inflammatory responses, and improved intestinal barrier function, all of which contribute to the restoration of disrupted gut microbiota (Figures 3 and 4). Subsequently, 16S rDNA sequencing analysis revealed that GEN significantly altered the gut microbiota, as shown by changes in β‐diversity and an increase in butyrate‐ and propionate‐producing bacterial communities. It should be noted that gut microbiota‐derived SCFAs (butyrate and propionate) play a crucial role in maintaining host immune composition and repair capacity post‐MI [5]. For example, the cardioprotective effects of fenofibrate against heart failure might be attributed to increased production of SCFAs [51]. Notably, correlation analysis between plasma SCFAs (butyric and propionic) and cardiac function indices (LVEF, LVFS, E'/A') revealed that both butyric and propionic were positively correlated with LVEF, LVFS, E'/A' (Figure 5). Taken together, these findings indicated that GEN's enhancements in gut microbiota might be a critical factor for its cardioprotective effects post‐MI.

The heart is extensively innervated by the autonomic nervous system, mainly composed of sympathetic and parasympathetic nerves, which form an exquisitely complex and varying network to regulate cardiac function [52]. After the ischemic injury, sympathetic axon fibres become infracted and degenerated, followed by heterogeneous patterns of neural remodelling within the scar [53]. Significantly, abnormal sympathetic regeneration and innervation could increase repolarization dispersion and accentuate the automaticity and trigger activity, which accounts for enhanced susceptibility to malignant arrhythmias [54]. Accumulating clinical studies have demonstrated that increased cardiac sympathetic activity is highly associated with poor prognosis and lethal VAs [55]. Therefore, reducing the sympathetic nerve activity might be a potential strategy to alleviate cardiac dysfunction and improve the prognosis of MI. Notably, previous studies have revealed that overexpression of proinflammatory cytokines in the PVN was highly associated with sympathetic activation and cardiac dysfunction [55]. In this study, we found that Gen could alleviate MI‐induced inflammation in the PVN, as evidenced by decreased levels of inflammatory cytokines (TNF‐α, IL‐1β), increased levels of anti‐inflammatory cytokine (IL‐10), and a lower percentage of activated microglia. Notably, sympathetic activation was also found to be associated with significant structural and functional alterations in the hippocampus, including a reduction in dentate gyrus (DG) volume, impaired neurogenesis, and increased astrogliosis [25]. Of note, our study demonstrated that GEN was effective in alleviating these hippocampal impairments by mitigating the reduction in DG volume, downregulating the expression of GFAP (a marker of astrogliosis), and upregulating the expression of BDNF and its receptor TrkB in the hippocampus. DG, as a subregion of the hippocampus, plays a critical role in neurogenesis and synaptic plasticity, and structural shrinkage of DG has been closely linked to heightened sympathetic activity and neuroinflammation. Restoration of DG volume suggested that GEN might promote neuronal survival or regeneration, potentially through its neuroprotective and anti‐inflammatory properties; Reduced BDNF and TrkB levels have been directly associated with impaired neuroplasticity, and sympathetic overactivation is known to suppress BDNF expression through mechanisms involving oxidative stress and inflammation. By restoring hippocampal BDNF and TrkB expression, GEN has the potential to counteract these deleterious effects, promoting hippocampal neurogenesis and synaptic plasticity; Furthermore, the decrease in GFAP levels observed following GEN treatment suggested a reduction in reactive astrogliosis, a pathological response frequently triggered by neuroinflammation and oxidative stress, which are central mediators of sympathetic overactivation [25]. Thus, GEN could alleviate the MI‐induced hippocampal impairments, thus inhibiting sympathetic activation post‐MI. More importantly, GEN significantly inhibited sympathetic activity, as demonstrated by decreased sympathetic activity indexes, including norepinephrine (NE) and TH, a critical enzyme responsible for generating NE. Collectively, these data indicated that GEN could exert inhibitory effects in inflammation in PVN and astrogliosis in hippocampus, which may contribute to the reduction of sympathetic activation and the enhancement of cardioprotective effects post‐MI.

Emerging evidence highlights a strong association between impaired gut–brain axis and cardiovascular diseases [56]. After the MI injury, sympathetic hyperactivity combined with intestinal pathological injury (congestion, ischemia, oedema, etc.) could result in a significant change in gut microbial metabolites, which are the pivotal interaction between gut bacteria and brain function [57]. Among many gut microbial metabolites, short‐chain fatty acids (SCFAs, < 6 carbons) such as butyrate and propionate, mainly produced by the dietary fibre fermentation in the colon, have been reported to exert potent cardiovascular protective effects due to their critical role in energy metabolism, parasympathetic activation, and oxidative stress resisting [58]. For example, Yu et al. reported that butyrate treatment remarkably reduces the myocardial ischemia/reperfusion (I/R) injury through a gut‐brain neural circuit, and the protective effects of butyrate are likely mediated via the inhibition of the sympathetic pathway [59]. In addition, Zhou et al. found that oral supplementation with propionate was shown to improve ventricular electrical remodelling at least by parasympathetic excitation based on the gut–brain axis post‐MI [60]. In this research, we found that GEN could significantly reverse the decreased expression of butyrate and propionate induced by MI, indicating that the improvement of GEN in restoring the gut–brain axis post‐MI is possibly mediated via the regulation of SCFAs.

There were still several limitations in the study. First, although GEN led to a remarkable alteration in gut microbiota and permeability post‐MI, we could not rule out the possibility that the beneficial effects of GEN depended on other pathways or mechanisms rather than the gut microbiome. Thus, in the following experiments, we need to investigate whether the depletion of gut microbes by antibiotic treatment could abolish the cardioprotective effects of GEN and whether faecal microbiota transplantation from MI + GEN mice to MI mice could attenuate cardiac pathology and function. Second, we did not figure out whether the alteration of neuroinflammation in the PVN was caused by the changes in gut microbes, suggesting that more evidence is needed to support the improvement of GEN on the impaired gut–brain axis. Third, the use of only male mice aged 8–10 weeks in this study might pose a limitation to the generalizability of the results. Therefore, future research will incorporate mice of diverse ages (both male and female) to gain a more comprehensive understanding of the mechanisms underlying MI. Additionally, given the limited persuasiveness of data from the mice model, further investigations are required to investigate the effects of GEN treatment in clinical application, providing significant clinical implications for patients after MI.

In conclusion, this study demonstrated that the cardioprotective effects of GEN were mediated possibly via improving the gut–brain–heart axis following MI. First, we found that GEN alleviated myocardial injury and improved heart function post‐MI. In addition, these effects might be associated with alterations in the gut microbiota, alleviation of neuroinflammation with PVN, and inhibition of sympathetic hyperexcitability. Mechanically, microbiota‐derived SCFAs might be the critical factor linking gut microbiota and neuroinflammation with PVN. Furthermore, reduced neuroinflammation with PVN contributes to inhibiting sympathetic activation, and a reduction in sympathetic activity plays a critical role in protecting the myocardium from MI injury. These data provide the experimental basis for the clinical application of GEN treatment in treating MI.

## Author Contributions

**Jie Chen:** conceptualization (equal), investigation (equal), writing – original draft (equal). **Tong Zhu:** conceptualization (equal), investigation (equal), writing – original draft (equal). **Jinbao Yang:** funding acquisition (equal), investigation (equal), methodology (equal). **Mengqing Shen:** methodology (equal), project administration (equal). **Danmei Wang:** investigation (equal), methodology (equal). **Boyuan Gu:** data curation (equal), formal analysis (equal). **Jin Xu:** funding acquisition (equal). **Mingxia Zhang:** validation (equal). **Xiuli Hao:** software (equal). **Zheng Tang:** supervision (equal). **Jie Tong:** visualization (equal). **Yan Du:** funding acquisition (equal). **Bao Zhang:** funding acquisition (equal), project administration (equal), writing – review and editing (equal). **Hongbao Li:** funding acquisition (equal), writing – review and editing (equal). **MengLu Xu:** funding acquisition (equal), writing – review and editing (equal).

## Conflicts of Interest

The authors declare no conflicts of interest.

## Supporting information


**Figure S1.** Identification of myocardial infarction in mice. (A) Representative image of electrocardiogram in Sham mice. (B) Representative image of electrocardiogram in MI mice.

## Data Availability

The datasets used and analysed in the current study are available from the corresponding author on reasonable request.
