# Supplementary material for: Geniposide Protects Against Myocardial Infarction Injury via the Restoration in Gut Microbiota and Gut–Brain Axis
Source: J Cell Mol Med. 2025 Feb 5;29(3):e70406. doi: 10.1111/jcmm.70406 (PMC11798748; doi:10.1111/jcmm.70406)
Supplement: Supplementary file 1 — Figure S1. Identification of myocardial infarction in mice. (A) Representative image of electrocardiogram in Sham mice. (B) Representative image of electrocardiogram in MI mice. [file JCMM-29-e70406-s001.docx]

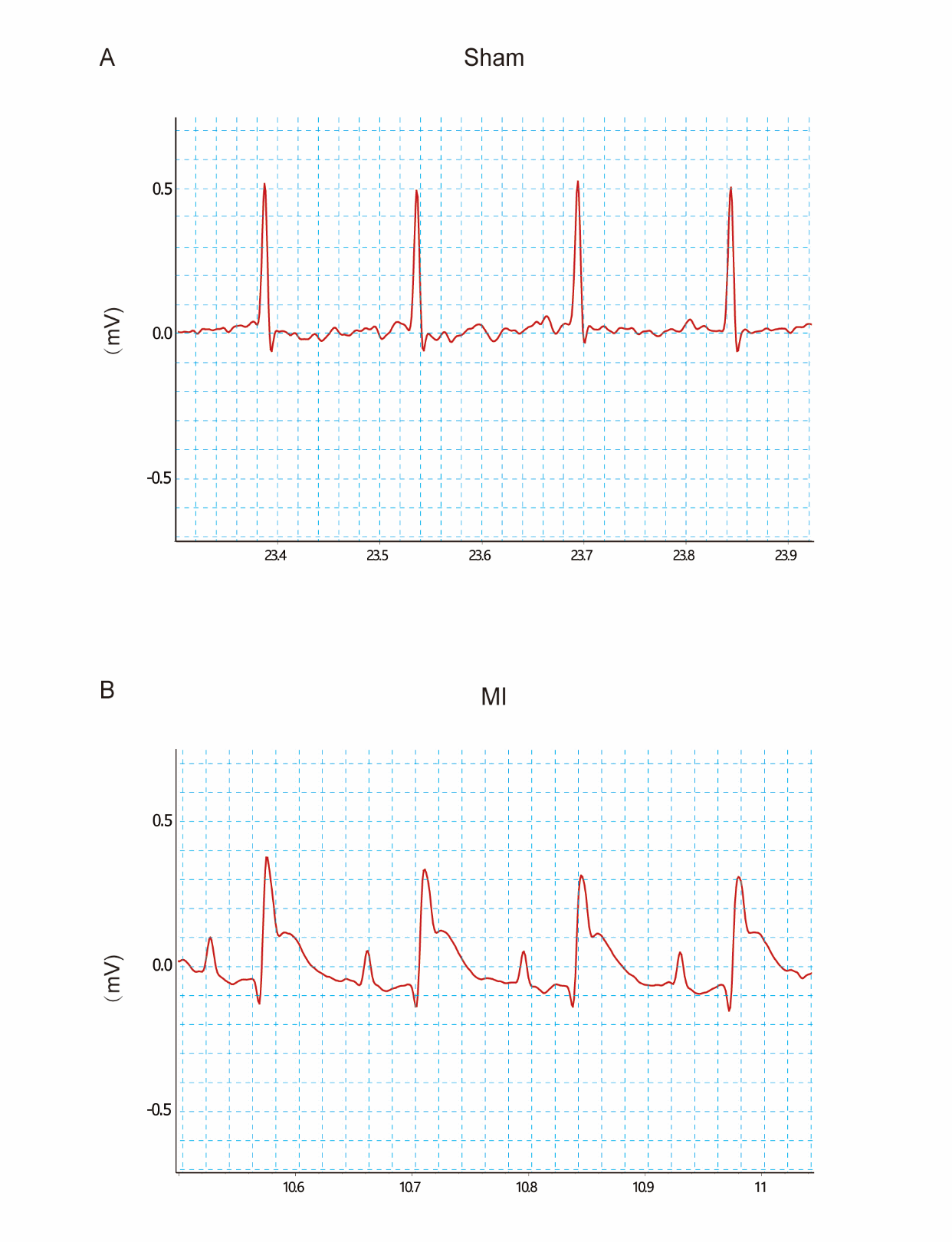


**Supplement Figure 1. Identification of myocardial infarction in mice.** (A) Representative image of electrocardiogram in Sham mice. (B) Representative image of electrocardiogram in MI mice.
